# Does gender matter? The association between different digital media activities and adolescent well-being

**DOI:** 10.1186/s12889-022-12670-7

**Published:** 2022-02-10

**Authors:** Robert Svensson, Björn Johnson, Andreas Olsson

**Affiliations:** 1 Department of Criminology, Malmö University, 205 06 Malmö, Sweden; 2 Department of Social Work, Malmö University, 205 06 Malmö, Sweden; 3 Department of Clinical Neuroscience, Division of Psychology, Karolinska Institutet, 171 77 Stockholm, Sweden

**Keywords:** Adolescents, Screen time, Social media, Playing games, Well-being

## Abstract

**Background:**

Previous research on the relationship between social media use and well-being in adolescents has yielded inconsistent results. We addressed this issue by examining the association between various digital media activities, including a new and differentiated measure of social media use, and well-being (internalizing symptoms) in adolescent boys and girls.

**Method:**

The sample was drawn from the four cross-sectional surveys from the Öckerö project (2016–2019) in eight municipalities in southern Sweden, consisting of 3957 adolescents in year 7 of compulsory education, aged 12–13. We measured the following digital media activities: playing games and three different activities of social media use (chatting, online sociability, and self-presentation). Our outcome measure was internalizing symptoms. Hypotheses were tested with linear regression analysis.

**Results:**

Social media use and playing games were positively associated with internalizing symptoms. The effect of social media use was conditional on gender, indicating that social media use was only associated with internalizing symptoms for girls. Of the social media activities, only chatting and self-presentation (posting information about themselves) were positively associated with internalizing symptoms. Self-presentation was associated with internalizing symptoms only for girls.

**Conclusion:**

Our study shows the importance of research going beyond studying the time spent on social media to examine how different kinds of social media activities are associated with well-being. Consistent with research in psychology, our results suggest that young girls posting information about themselves (i.e. self-presentation) might be especially vulnerable to display internalizing symptoms.

## Introduction

Adolescents of today spend an increasing portion of their free time using different digital media, such as smartphones, computers, and tablets [1]. In Sweden, the proportion of 14-year old adolescents using the internet for more than 3 h/day increased from 30% in 2010 to 76% in 2020 [2], and in the US, 45% of teens report that they are online on a “near-constant” basis [3]. At the same time, the rate of depression, anxiety, and suicide among adolescents has risen to historically high levels over the past 10–15 years [4–6]. For example, the level of depression in 10–17-old Swedish girls has surged from approximately 500 to more than 1.000 per 100.000 from 2008 to 2018 [7]. Although the reasons for this increase in depression rate are likely to be multifaceted, including changed diagnostic criteria, the use of digital media has attracted increasing attention as a potential causal factor [8].

The association between digital media use (e.g., playing games, social media use, watching TV) or social media use more specifically (e.g., different activities on Instagram, Facebook or Snapchat) and well-being has been examined (and debated) [9, 10] in several studies with mixed results [11–13]. Whereas the majority of studies – many of which have been based on cross-sectional designs – have found a negative association [8, 11], the few available longitudinal studies have shown either that social media use predicts lower well-being in terms of depression and internalizing symptoms (e.g., depression, anxiety, hypersensitivity, headache, worry) [14–17], no association [18–20], or that depression predicts an increase in social media use [21, 22]. In addition, some investigators have found evidence of a curvilinear u-shaped association [23, 24]. Other research has examined potential mediators, and found that the association between social media use and well-being could be mediated by factors, such as cyberbullying, lack of sleep, and lower physical activity [15].

An intriguing possibility is that the inconsistent results in the existing literature are related to key methodological differences between studies, limiting the generalizability and conclusions that can be drawn from them [1]. To move beyond these limitations, we performed a large cross-sectional study and used in-depth measures of social media activities and internalizing symptoms, which constitute an important aspect of mental well-being. Our goal was to provide a deeper understanding of, not only where on the internet adolescents spend their time, but what kind of activities might contribute to changes in mental well-being. To clarify the specific contributions of our study, we begin by listing three central limitations in existing research.

A first limitation in past research that might contribute to the mixed findings is the lack of interest in specific digital media activities, and to what degree they are differently associated with well-being [18, 25, 26]. For example, studies that do not differentiate clearly between various digital media activities, have found both negative [27], and non-existent associations with well-being [18]. Internet use can include a wide range of activities that differ across gender in terms of agency, display mode, and social meaning. Therefore, it is important to examine different activities separately. A number of studies have found that playing games is associated with lower well-being in terms of internalizing problems and depressive symptoms [28–31]. Importantly, social media use was found to be more strongly associated with well-being than playing games [1, 14]. One explanation of this difference could be that social media presents more opportunities for social comparison, which is known to decrease well-being, in comparison to gaming [1].

A second limitation in past research that might contribute to the mixed findings, is the varying measures used to assess social media use more specifically. For example, adolescents have been asked to define social media themselves [32], and self-report how active they have been on different platforms [20, 33], whether they chat or interact with their friends on different platforms [17, 34], and use a combined measure of instant messaging, photo-sharing or other social media activities on different platforms [22]. Different means of assessing social media might tap into distinct activities with varying relevance for the development and expression of well-being. We argue that it is important to go beyond existing approaches to examine both how and in what way different social media activities are associated with well-being in boys and girls separately.

To address these open questions, our study focuses on three different social media activities: (i) chatting in real-time with friends on platforms that are “private” (one-on one or private group chats); (ii) online sociability, i.e. communication taking place in public social media that can be viewed by others (not necessarily in real time); and (iii) self-presentation, such as selfies, film clips or other types of personal information that can be viewed by others. As far as we know, no previous study has examined how these three activities are associated with well-being for boys and girls. Self-presentation, which has been highlighted as a key factor underlying the association between social media use and adolescent well-being [35–38], indicates an individual’s motivation to “brand” themselves and to reach digital social status through likes and new followers [39]. This search for social rewards lays the ground for social comparisons, known to affect well-being negatively [35, 40]. Indeed, recent meta-analyses [41, 42] demonstrate that social comparison in general, and upwards social comparison (i.e. comparison with superior other) in particular, predicts a decrease in well-being [35, 39, 43]. The lack of positive feedback (i.e. social rewards) and the presence of social punishment from the social media community has been shown to have a negative influence on the adolescent’s well-being [13, 44]. In light of these lines of research, we assume that a higher reliance on self-presentation renders the individual more vulnerable to negative feedback.

A third limitation in previous research is that previous studies have treated gender as a control variable, overlooking the possibility that the association between digital media use and well-being is different for boys and girls [14, 26]. For example, some researchers have analysed boys and girls together, founding that digital media use was not associated with well-being [20], whereas others have shown girls to have lower well-being as indicated by, for example, depressive symptoms [6, 37, 45], anxiety [6, 45] and internalizing problems [16]. Girls use social media [1, 2, 28, 46] and chat with friends [28] more than boys who in turn tend to spend more time gaming [1, 2, 28]. In addition, girls more frequently post different types of selfies, use filters, manipulate their photos, and delete post more frequently than boys, whereas boys update their profile more often on sport and technology [2, 37, 43, 47, 48]. Against this background we argue that it is important to examine the associations by gender.

The importance of including gender as a variable is supported by previous research finding social media use to be more strongly associated with well-being for girls than for boys [1, 17, 49, 50], whereas some did not find any gender differences [16]. Differences in the selfie culture may be one explanation that helps us to understand why the association between self-presentation and depressive symptoms is larger for girls [37]. Gaming has been found to be associated with well-being for both boys and girls [1]. These gender differences indicate that social media use, and different activities of social media may have a larger association with well-being for girls, and that the association between gaming and well-being might be rather similar for boys and girls.

To sum up, we identified three areas of limitations in previous research related to the importance of separately examining the role of (1) kinds of digital media activities, (2) different types of activities within social media use, and (3) gender. In our cross-sectional study, we aim to use various measures of digital media activities, well-developed assessments of social media activities, and examine how these activities are associated with internalizing symptoms as a measure of girls’ and boys’ well-being. In spite of the inherent limitations of cross-sectional designs, which are unsuitable to make strong causal claims, we believe that these methodological improvements will contribute to our understanding of the specific processes linking social media use and mental health problems and well-being in adolescents.

We test the following three hypotheses:

- H1: Different digital media activities are differently associated with internalizing symptoms: (a) social media use will be positively associated with internalizing symptoms, and (b) playing games will be positively associated with internalizing symptoms, but the association will be stronger for social media use compared to playing games.
- H2: Different social media activities, such as (a) chatting, (b) online sociability and (c) self-presentation, will all be positively associated with internalizing symptoms.
- H3: Social media use and different activities of social media use, such as (a) chatting, (b) online sociability and (c) self-presentation will be more strongly associated with internalizing symptoms for girls than for boys. The association between playing games and internalizing symptoms will not differ between girls and boys.

## Method

### Study design

The data used are based on secondary data analysis from four cross-sectional surveys from the Öckerö project [51, 52], an evaluation of an alcohol prevention program [53]. The project included an annual self-report survey conducted in 17 secondary schools in eight small municipalities in the county of Skåne, Sweden. The survey was conducted in all classes in years 7–9 (i.e. 12–15 years of age), the final 3 years of compulsory education. The survey was conducted at the beginning of the autumn term in each of four successive years, 2016–2019.

### Participants

In this study, we employ data on youths in year seven (on average 13 years of age) year 2016, 2017, 2018 and 2019 from the Öckerö project. The study constitutes a census of 4256 adolescents. Following listwise deletion of missing values, the analyses below are based on 3957 respondents (50.2% boys). The non-responses were fairly evenly distributed across the included variables.

### Data collection

The data were collected through an online questionnaire that was introduced to the class members by researchers and assistants working on the project. The questionnaire was completed during lesson time and took an average of 30 min. Before completing the questionnaires, the students were given detailed information about the purpose of the survey. They were also informed that their participation was voluntary and anonymous. The research design and study procedures were approved by The Regional Ethical Review Board in Lund.

### Measures

#### Dependent variable

*Internalizing symptoms* scale was based on an additive index consisting of six statements: (1) I often feel sad and depressed, (2) I often worry about the future, (3) I often feel anxious and worried, (4) I often have a stomach ache or headache, (5) I often feel lonely, (6) I have difficulty sleeping and eating (response alternatives: totally disagree / disagree / agree / totally agree). The items used in this scale have carefully been chosen and measure emotional responses to stressors that are inwardly directed [54] and are similar to the emotional symptom dimension of the SDQ scale [55]. The scale has an alpha value of .87. This scale has previously been used in the study of alcohol use [51]. Similar measures have been used in previous research [16]. High scores indicate that the respondents have high level of internalizing symptoms.

#### Independent variables

*Social media use* is an additive index based on three items. *How often do you use a computer, mobile phone or tablet to do one of the following activities?* (1) Talk to friends at Skype, Kik, Viber, Whats’s app or similar – referred to as *Chatting*, (2) Stay in contact with and stay informed about my friends via Facebook, Instagram or similar – referred to as *Online sociability*, (3) Post information about myself on Facebook, Instagram, Snapchat or other social media – referred to as *Self-presentation*. Response alternatives: never / about once a month / about once a week and / several times a week / every day. The three measures of social media use will also be analysed as separate measures of social media in a supplemental regression model. Although, chatting and online sociability seems to be rather similar, we argue that it will not be a problem of using them as distinct components (*r* = .36). The measures will be treated as continuous variables in the following analyses.

*Playing games* is a single item based on *How often do you use a computer, mobile phone or tablet to do one of the following activities?* (1) Play games. Response alternatives: never / about once a month / about once a week and / several times a week / every day. The measure will be treated as a continuous variable in the following analyses. These items of digital activities have been used in previous studies [52].

*Demographic variables* are represented using two different measures in the analyses. *Gender* is coded as 0 for girls and 1 for boys. *Country of birth* is coded as 0 if the respondent is born abroad and 1 if the respondents is born in Sweden. Sweden is a country with a high proportion immigrant from non-European countries and therefore we decided to adjust for country of birth.

*Year* represents the year when the study was conducted and is used as three dummy variables. Year 2016 is the reference category in the analyses and year 2017, 2018 and 2019 are the other years involved in the analyses.

For a description of the measures used see Table 1.

**Table 1:** Descriptive statistics (*N* = 3957)

	Mean/%	SD	Min	Max
Internalizing symptoms	10.51	4.33	5	24
Social media use	10.14	3.07	3	15
Chatting	3.78	1.39	1	5
Online sociability	3.83	1.35	1	5
Self-presentation	2.54	1.36	1	5
Playing games	3.57	1.30	1	5
Boys	50.2%	–	0	1
Born in Sweden	86.8%	–	0	1
Year 2016	25.2%	–	0	1
Year 2017	24.1%	–	0	1
Year 2018	24.8%	–	0	1
Year 2019	25.9%	–	0	1

#### Statistical analyses

First, we compared the mean values of internalizing symptoms, social media use, and the three dimensions of social media use, and playing games by gender. We also calculated the effect size measure of Cohen’s *d*. Second, we estimated a number of ordinary least squares regression models. In the first model, we included social media use, playing games and gender. As curvilinearity could be a problem in interaction models [56, 57] and since previous research have found evidence of curvilinear associations [9, 23], we also test for curvilinear relationships and added squared terms of social media use and playing games to the linear model. In the second model, we included several interaction terms of gender × social media use and gender × playing games. In this model we also added our squared terms of social media use and playing games. In all the regression models we adjust for country of birth, year of study, the interaction between gender × year and dummies of the 17 schools. Finally, all the regression models were also estimated using the three different dimensions of social media use, i.e. chatting, online sociability and self-presentation. In these models we also test for curvilinear relationships by adding squared terms. The continuous variables were mean-centered prior to their inclusion in interaction terms. Finally, to illustrate the interactions, we plotted the interaction using the predictive margins plot command in Stata. All analyses were conducted in Stata/SE version 13.1.

## Results

Table 2 presents differences between girls and boys in relation with internalizing symptoms, social media use, chatting, online sociability, self-presentation and playing games. We observe that girls report significantly higher on the internalizing symptoms than boys (*t* = 19.99, *p* = < .001). Moreover, girls score significantly higher on the measure of social media use (*t* = 11. 47, *p* = < .001), as well as on chatting (*t* = 5.14, *p* = < .001), online sociability (*t* = 9.49, *p* = < .001) and self-presentation (*t* = 11.01, *p* = < .001). Boys report significantly higher on the playing game measure (*t* = − 28.81, *p* = < .001). The effect sizes presented by the Cohen’s *d* range from .16 (for online sociability) to −.92 (for playing games).

**Table 2:** Internalizing symptoms and digital activities by gender, independent *t*-test and effect sizes (Cohen’s *d*) (*N* = 3957)

	Girls		Boys				
	Mean	SD	Mean	SD	*t*-test	*p*-value	*d*
Internalizing symptoms	11.83	4.58	9.20	3.63	19.99	<.001	.64
Social media use	10.70	2.90	9.60	3.13	11.47	<.001	.36
Chatting	3.90	1.35	3.67	1.43	5.14	<.001	.16
Online sociability	4.03	1.28	3.63	1.39	9.49	<.001	.30
Self-presentation	2.77	1.37	2.30	1.32	11.01	<.001	.35
Playing games	3.03	1.29	4.11	1.07	−28.81	<.001	−.92

In order to examine our key hypotheses, i.e. whether the different digital media activities are differently associated with internalizing symptoms for girls and boys we present a number of regression models in Table 3. The results presented in model 1 show that social media use and playing games are both positively associated with internalizing symptoms. According to the standardized regression coefficient, social media use is slightly more strongly associated with internalizing symptoms than playing games. The results also show that the squared terms of the two measures are significant, indicating a curvilinear association between both digital media activities and internalizing symptoms. Gender is negatively associated with internalizing symptoms, which means that girls report higher on internalizing symptoms, adjusting for the other predictors.

**Table 3:** The relationship between internalizing symptoms and digital activities (social media use and playing games). Ordinary least square regression

	Model 1			Model 2		
	*B*	Beta	*p*-value	*B*	Beta	*p*-value
Social media use	.191	.135	<.001	.288	.204	<.001
Social media use^2^	.032	.088	<.001	.027	.075	<.001
Playing games	.320	.096	<.001	.294	.088	.002
Playing games^2^	.143	.060	.001	.139	.058	.003
Boy	−2.389	−.278	<.001	−2.343	−.270	<.001
Social media use × Boy				−.191	−.098	<.001
Playing games × Boy				.072	.013	.521
Controls ^a^	Yes			Yes		
R^2^	.129			.133		
N	3957			3957		

Note: *B* = unstandardized coefficient; Beta = standardized coefficient. The *p*-values are calculated using robust standard errors. ^a^ Control variables included in the models are country of birth, year of study measured as three dummies, gender × year of study, and the different schools are included as 17 dummies

In the second model, where the interaction terms included, and the results show that the interaction between gender and social media use is strongly negatively associated with internalizing symptoms (*B* = −.191, *p* = < .001), meaning that the association between social media use is more pronounced for girls then for boys. The squared term of social media use is still associated with internalizing symptoms, indicating that the association is not linear it is curvilinear. Finally, the interaction between gender and playing games is not significantly associated with internalizing symptoms, i.e. there is no gender differences in the association between playing games in relation with internalizing symptoms.

**Fig. 1:**
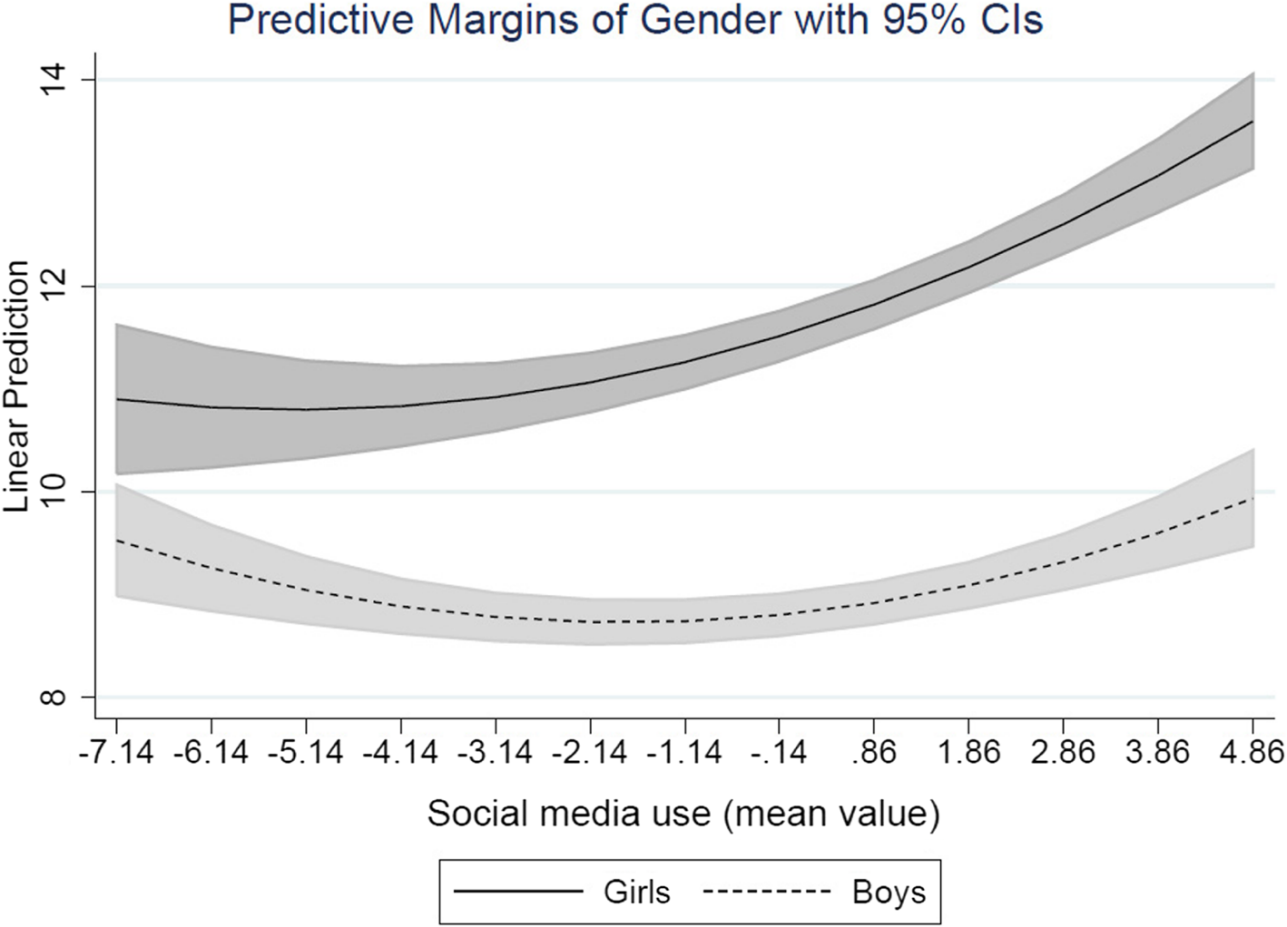
Interaction between gender and social media use (low value = seldom; high value = often) in the association with predicted values of internalizing symptoms (high value = more symptoms)

To visualize the interaction, we plotted the predicted values of internalizing symptoms on social media use by gender, with their 95% CIs. Figure 1 show that the association between social media use and internalizing symptoms is only significant for girls but not for boys. The association is curvilinear indicating that the association between social media use and internalizing symptoms becomes more and more pronounced the more girls are using the social media.

Finally, we estimated the regression models using the three different dimensions of social media use – chatting, online sociability and self-presentation in the association with internalizing symptoms in Table 4. Of the three social media activities, were chatting and self-presentation positively associated with internalizing symptoms, whereas online sociability is negatively associated with internalizing symptoms, adjusting for the other factors presented in model 1. According to the standardized regression coefficients, the results shows that self-presentation is the activity that is most strongly associated with internalizing symptoms of the different digital activities. Further, the results in model 2, show that self-presentation interact with gender in the association with internalizing symptoms (*B* = −.459, *p* = < .001), indicating that self-presentation is significantly stronger associated with internalizing symptoms for girls than for boys. The results also show that the interaction between gender and online sociability is near significant (*B* = −.215, *p* = .057). This interaction indicates that online sociability is negatively associated with internalizing symptoms for boys.

**Table 4:** The relationship between internalizing symptoms and different social media activities and playing games. Ordinary least square regression

	Model 1			Model 2		
	*B*	Beta	*p*-value	*B*	Beta	*p*-value
Chatting	.300	.096	<.001	.287	.092	.002
Chatting^2^	.094	.049	.017	.094	.050	.016
Online sociability	−.159	−.050	.004	−.035	.011	.696
Self-presentation	.427	.134	<.001	.644	.203	<.001
Playing games	.305	.091	<.001	.262	.079	.007
Playing games^2^	.140	.059	.001	.129	.054	.006
Boy	−2.339	−.270	<.001	−2.309	−.266	<.001
Chatting × Boy				.045	.011	.656
Online sociability × Boy				−.215	−.049	.057
Self-presentation × Boy				−.459	−.100	<.001
Playing games × Boy				.078	.014	.529
Controls ^a^	Yes			Yes		
R^2^	.133			.141		
N	3957			3957		

Note: *B* = unstandardized coefficient; Beta = standardized coefficient. The *p*-values are calculated using robust standard errors. ^a^ Control variables included in the models are country of birth, year of study measured as three dummies, gender × year of study, and the different schools are included as 17 dummies

**Fig. 2:**
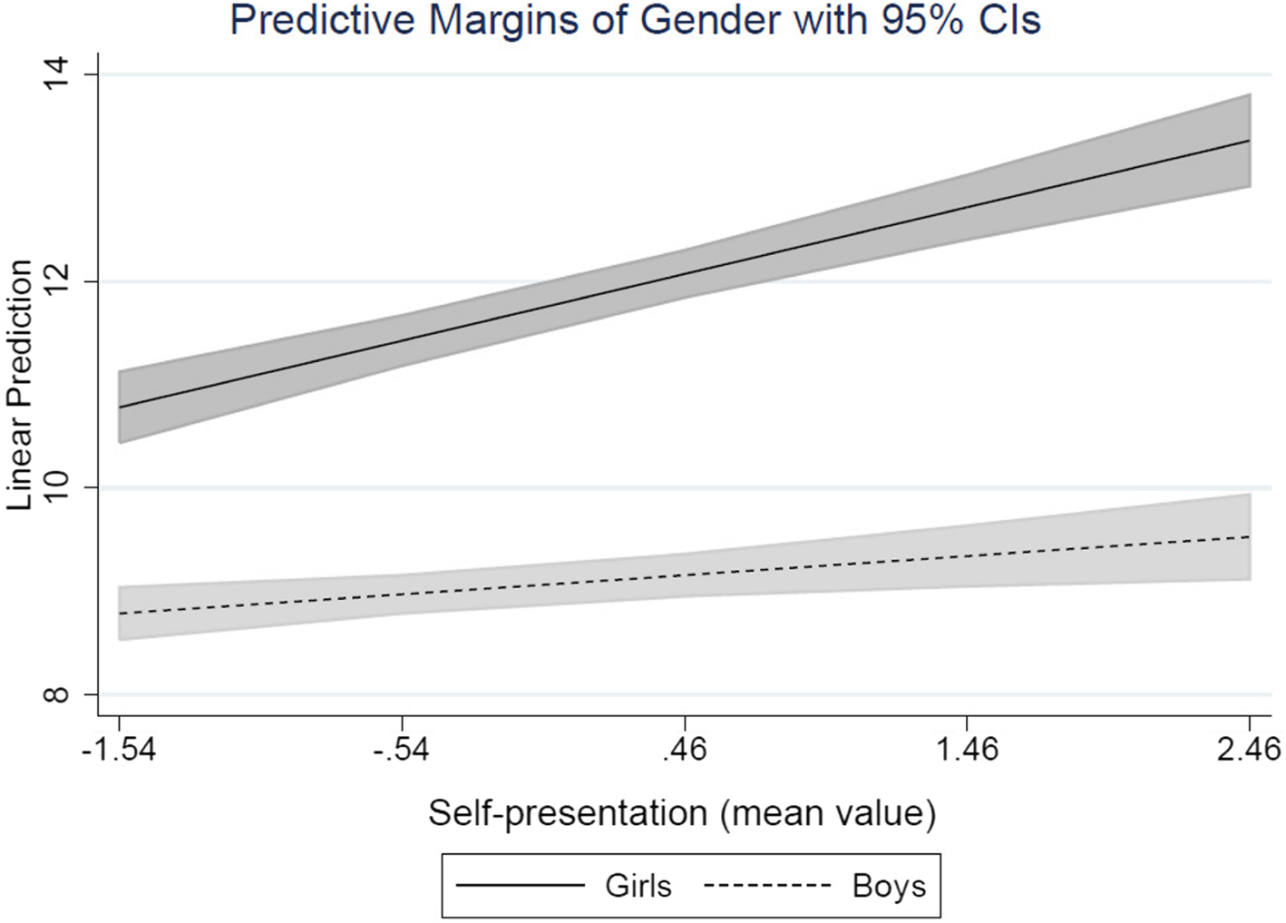
Interaction between gender and self-presentation (low value = seldom; high value = often) in the association with predicted values of internalizing symptoms (high value = more symptoms)

To visualize the interaction between self-presentation and gender, we plotted the predicted values of internalizing symptoms. Figure 2 show that the association between self-presentation and internalizing symptoms is only significant for girls but not for boys.

## Discussion

The current study examined how different digital activities (social media use and playing games), and different types of social media use (chatting, online sociability and self-presentation) are associated with internalizing symptoms as a measure of well-being in adolescent boys and girls. Our primary finding was that social media use was strongly and positively associated with internalizing symptoms in girls. This finding dovetail with previous research highlighting the negative health effects of social media use that offers an arena for self-presentation and self-comparison. Interestingly, the association in our study showed a curvilinear association, suggesting that the association will become stronger as the use of social media increases. The different activities of social media use were differently associated with internalizing symptoms. On the one hand, we found both chatting and self-presentation to be positively associated with internalizing symptoms and demonstrated that self-presentation was more strongly associated with internalizing symptoms for girls. On the other hand, online sociability was negatively associated with internalizing symptoms and this pattern was more pronounced for boys. Finally, our results also indicated that playing games was positively associated with internalizing symptoms.

Our results are consistent with previous cross-sectional [28] and longitudinal [15] studies that found social media use to be more strongly associated with measures of well-being for girls than for boys. At the same time, our results contradict findings from other longitudinal studies that found no association between social media use and well-being [18–20]. A limitation in some of these longitudinal studies is that they used gender as a control variable and not examined whether the association could be different for boys and girls [20, 22]. Another limitation of previous studies is that varying measures were used to assess social media use. For example, several researchers [22] combined a number of different social media activities within a combined measure of social media use, and others [18] used a measure of social media asking how much time the adolescents spend on social networking (e.g., Facebook) on a typical day.

A key finding in our study is that self-presentation is the social media activity associated with internalizing symptoms for girls. This result is in line with the argument that adolescents that present information about themselves, often in search of likes and followers, will be more susceptible to self-comparisons [35, 39–42], which is known to lead to decrease in well-being [37, 41, 42]. Additionally, not receiving the social rewards one hoped for (e.g., likes and followers), and the presence of social punishers, might be linked to an experienced loss of self-worth, stress and social isolation, all of which are known features of internalizing symptoms [35, 39, 43]. Finally, very few studies have gone beyond establishing an association to trying to explain *why* there is an association between social media use and well-being [35].

Our study improved on several methodological shortcomings in previous research studying the relationship between social media use and well-being. In particular, we measured (1) both social media use and playing games, (2) different forms of social media use, including chatting, online sociability and self-presentation, which has not been used before in the study of adolescent’s well-being, and (3) examined the role of gender.

A few limitations also need to be acknowledged. First, our cross-sectional design constrained our ability to draw any causal conclusions. For example, social media might cause internalizing symptoms, but it is also possible that internalizing symptoms influence social media use and finally, the association can be bi-directional. Our results do, however, closely align with the growing recognition of the role of social media in negatively affecting psychological well-being [35, 41, 42]. A second weakness of our study is that the response options in our survey did not allow for the same resolution in terms of time (number of hours) spent with social media as several previous studies [1, 14], although a few previous studies have used similar response alternatives as ours [15, 33]. This indicates that we cannot determine the exact number of hours that the respondents spend on social medial on a daily basis. That being said, our results nonetheless indicate the importance for future studies to employ more detailed measures of time spend online. Finally, our gaming measure includes both gaming together with other people and gaming alone, making it impossible to draw conclusions about the role of socialization during gaming.

## Conclusion

In conclusion and in line with our hypotheses, our study shows that different social media activities are differently associated with internalizing symptoms as a measure of adolescent’s well-being, and that activities entailing self-presentation stand out because of their negative association with well-being for girls, but not boys. Our results suggest that policy makers should be aware of that not all social media activities are created equal in terms of their implications for the health of adolescents, and that these implications might be gender-specific. Future research needs to continue to use increasingly differentiated measures of social media activities, and include both longitudinal and experimental designs to provide a better understanding of how these activities are causally linked to the health of the young.

## Data Availability

The datasets used in the current study are not publicly available due to restrictions made by the Regional Ethical Review Board in Lund, Sweden, but are available from the corresponding author on reasonable request.
